# Recommendation to improve chronic kidney disease management guideline in primary healthcare, KwaZulu-Natal

**DOI:** 10.4102/curationis.v48i1.2623

**Published:** 2025-02-25

**Authors:** Verosha Ramkelawan, Pretty N. Mbeje, Ntombifikile G. Mtshali

**Affiliations:** 1 School of Nursing and Public Health, College of Health Sciences, University of KwaZulu-Natal, Durban, South Africa

**Keywords:** KwaZulu-Natal, South Africa, management guidelines, guidelines awareness, referral process, chronic kidney disease, primary healthcare

## Abstract

**Background:**

The prevalence of chronic kidney disease (CKD) is high in KwaZulu-Natal (KZN), especially in individuals with risk factors such as HIV, diabetes mellitus and hypertension. Despite existing guidelines, the study identified multifactorial gaps in implementing CKD management measures at the primary healthcare (PHC) level. This leads to late-stage diagnoses and increased burdens on tertiary institutions, as identified in institutional statistics. The study was therefore conducted in four different settings: a tertiary hospital in Durban and three PHC centres across KZN (two urban centres and one in a township).

**Objectives:**

The study’s objective is to describe the perceptions of healthcare professionals on the management of CKD, in the PHC setting in KZN, South Africa.

**Method:**

A qualitative, exploratory design involving healthcare professionals was adopted. Purposeful sampling was used. Open coding and conventional content analysis were adopted to analyse data.

**Results:**

Findings revealed three overarching themes: (1) knowledge and awareness about existing guidelines, (2) fragmented system and a lack of clear guidelines and (3) implications of clear guidelines on patient care. Participants demonstrated diverse awareness of guidelines, revealing a need for continuous education. Participants expressed needs for continuous education and clear guidelines.

**Conclusion:**

The study highlights an urgent need to bridge the knowledge gap and establish a cohesive healthcare system to address the growing CKD burden in KZN effectively.

**Contribution:**

The study emphasises the potential benefits of implementing clear guidelines to improve patient outcomes, early detection and appropriate interventions, thereby reducing the burden on tertiary facilities.

## Introduction

According to a systematic review and meta-analysis, the average prevalence of chronic kidney disease (CKD) on the African continent was 15.8%, with sub-Saharan Africa (SSA) having the highest prevalence of 17.7%, with almost 5% suffering from moderate or severe CKD (Winkler 2021). Higher preventive, detection and treatment strategies are needed to lower the risk of cardiovascular disease and the development of CKD-stage V, according to earlier research that indicated high prevalence rates of CKD in SSA, especially in West and Southern Africa (Winkler 2021). Patients in KwaZulu-Natal (KZN) are at increased risk of developing kidney disease as a result of the rising incidence of HIV infection, diabetes mellitus, hypertension, infectious diseases, low socioeconomic status and limited availability of preventive measures (Marie Patrice et al. 2019). Moodley et al. (2018) documented that at 20%, KZN is the province with the second-highest number of patients on dialysis therapy in South Africa.

Chronic kidney disease is defined as alterations in kidney function or structure, damage to the kidney or decreased glomerular filtration rate (GFR) of < 60 mL/m^2^/min for more than 3 months (Shlipak et al. 2021). Several people with CKD have non-specific symptoms or are asymptomatic at the onset of the disease (Tangri et al. 2021). Therefore, the diagnosis of CKD frequently occurs at CKD-stage V, as symptoms and complications such as anaemia, fluid overload and uraemia become apparent only in the later stages of CKD. On a global scale, self-control behaviours play a significant role in decelerating the progression of CKD among patients who do not necessitate dialysis. Caregivers for these patients should be selected to identify critical strategies to help patients take control of their health and delay the progression of CKD (Ayat Ali et al. 2021). However, nurses and doctors are also responsible for screening and managing CKD in the primary healthcare (PHC) setting and referring appropriately for further management to the patient’s base institution. Subsequently, patient care necessitates escalation to regional and tertiary healthcare institutions, where dialysis initiation occurs, and the patient undergoes comprehensive assessment and integration into the CKD programme. This includes the prompt diagnosis and efficient treatment of CKD, with the goals of reducing the disease burden, raising awareness and providing thorough health education to encourage disease prevention and slow or stop disease progression (Chironda & Bhengu 2019).

Chronic kidney disease contributes to global illness and exacerbates cardiovascular and all-cause fatality (Himmelfarb & Sayegh 2010). Chronic kidney disease disproportionately affects lower socioeconomic individuals with limited treatment access, making early identification and management crucial for equity (Chironda & Bhengu 2019). Proteinuria and GFR screening are cost-effective measures that enable early diagnosis and intervention to reduce the risk of progression to CKD-stage V, cardiovascular events and mortality (Molaoa, Bisiwe & Ndlovu 2021). Paget et al. (2015) recommended more frequent monitoring of kidney function in CKD patients, particularly those with a GFR below 60 mL/min/1.73 m^2^ and risk factors for rapid GFR decline, such as the use of non-steroidal anti-inflammatory drugs (NSAIDs). In addition with cases of abnormal kidney imaging (e.g. cystic kidney disease), persistent proteinuria with or without haematuria, or eGFR < 60 mL/min/1.73 m^2^ (recommended) or < 30 mL/min/1.73 m^2^ (obligatory), it is advisable to warrant a referral to a nephrologist (Paget et al. 2015).

There are approximately 2.3 nephrologists per million people in South Africa, which is overwhelming, way below the global norm, and tremendously insufficient to cover the nephrology treatment needs in South Africa (Wearne, Okpechi & Swanepoel 2019). The 2019 Global Kidney Health Atlas provided up-to-date data on global nephrology personnel, revealing a median density of 9.95 per million population (pmp) worldwide (Hassen et al. 2020). The South African Nursing Council (SANC) statistics in 2022 had a total of 227 nurses in the register who were registered with a qualification in nephrology nursing or urology nursing (SANC 2022) in a country with a population of more than 62 million (Department of Statistics SA 2022). The statistics showing a limited number of nurses with specialisation in nephrology nursing strengthened the case for a PHC approach in managing individuals with CKD.

South Africa’s guidelines emphasise the importance of competent screening and timely management of CKD patients, including reviewing prior blood results for electrolytes, urea and serum creatinine discrepancies. Paget et al. (2015) stated that individuals at increased risk of CKD should be screened annually, with interventions including blood pressure and glucose monitoring, urine dipstick testing and estimation of GFR. Furthermore, CKD has a gradual progression, with most patients going undetected and remaining asymptomatic or presenting with manifestations such as fatigue, swelling, weight loss and abdominal discomfort (Bovijn 2017).

Moreover, the prevention and management of CKD should focus on treating modifiable risk factors to reduce disease burden, decrease mortality rates and reduce healthcare costs (Jo et al. 2020). However, CKD is diagnosed at advanced disease stages, and the commencement of efficient interventions is delayed or missed (Tangri et al. 2021). Evidence suggests a failure to implement these interventions for several reasons. Bovijn documented that patients with abnormal laboratory results should be referred to a specialist earlier for prompt detection of CKD, thereby retarding its progression (Bovijn 2017). However, this rarely happens. Based on the above, the focus should be on competent screening and timely management of CKD, including a review of prior blood results for crucial electrolytes, blood urea and serum creatinine discrepancies to detect previous kidney dysfunction (Paget et al. 2015). This creates substantial responsibility for primary care practitioners; nevertheless, the above-mentioned interventions can be implemented at the PHC setting and community level.

### Problem statement

Because only 20% of those needing kidney replacement therapy can receive it in South Africa, early detection and prevention of CKD-stage V are essential at the PHC and community levels (Paget et al. 2015). Healthcare professionals need to understand that postponing the diagnosis and management of early stages CKD by a year can deteriorate the condition by 40%, raise the incidence of needing a kidney transplant or long-term dialysis treatment by 63% and exacerbate the risk of cardiovascular events by 8% (Tangri et al. 2023).

However, early identification of individuals at high risk for acquiring CKD remains poor (Bovijn 2017). Furthermore, it is documented that in SSA, three-quarters of adult patients die after being managed on dialysis treatment as a result of late entry into healthcare facilities, poor quality of dialysis and termination of dialysis because of exorbitant costs (Etheredge & Fabian 2017). The researcher’s discovery of this multifactorial gap in the earlier implementation of CKD care guidelines at the PHC setting in KZN served as the impetus for this investigation. Previous studies have also documented a gap in integrated information on the development and implementation processes and framework of the PHC Clinical Practice Guidelines in South Africa (Kredo et al. 2017).

This gap leads to the fragmentation of services from the PHC level to the regional level, a need for more awareness of the use of the referral system and unclearly defined roles. As a result, patients are often diagnosed with CKD at a late stage or even when they have already reached CKD-stage V, which then compromises their care. This has led to an increased load of patients with CKD-stage V and dialysis requirements in tertiary institutions (Mbeje 2022).

Therefore, this qualitative research study aims to improve the implementation of guidelines for CKD management at PHC setting in KZN, South Africa. The article describes healthcare professionals’ perceptions and understanding of the guidelines available for CKD management at PHC setting in KZN.

### Purpose of the study

**FIGURE 1:**
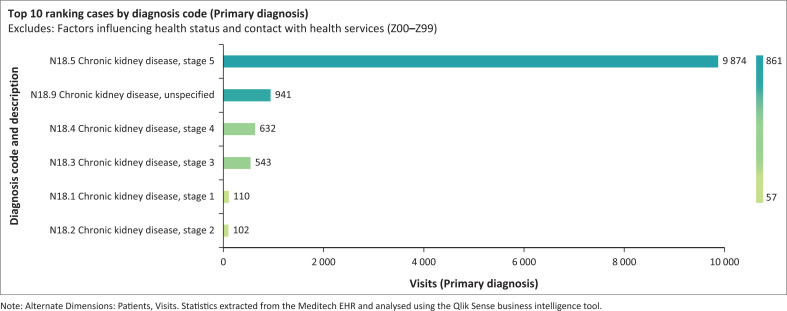
Electronic health record reflecting the increased number of visits at tertiary institutions at later stage chronic kidney disease.

**FIGURE 2:**
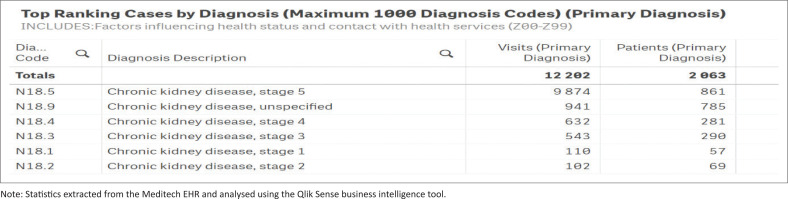
Electronic health records reflecting the increased number of patients at tertiary institutions at later stages of chronic kidney disease.

This study aims to enhance CKD management at PHC setting in KZN by identifying the main obstacles to the early implementation of CKD management recommendations. Once these factors are identified, policymakers can develop strategies to close these gaps, thereby limiting the burden of this disease on tertiary healthcare institutions. The study was initiated in response to the high influx of patients presenting with advanced-stage CKD at the tertiary institutions. This trend was confirmed at the tertiary institution by statistical data extracted from the Meditech electronic health record (EHR) system and analysed using the Qlik Sense business intelligence tool (see Figure 1 and Figure 2).

### Research objective

This study aimed to describe the perceptions of healthcare professionals on the management of CKD at the PHC setting in KZN, South Africa.

## Research methods and design

### Research approach

A qualitative, exploratory design was used to explore the lived experiences of healthcare professionals on CKD management at PHC setting in KZN. This approach allowed for an in-depth understanding of people’s subjective experiences and their perceptive processes by identifying key factors inhibiting the implementation of guidelines for managing CKD in the early stages. This enabled the researcher to achieve the goal of unpacking rich information on managing CKD at the PHC level. It was discovered that these inhibitors are multifactorial.

### Research setting

The research setting defined the choice of participants, who were healthcare professionals. The study was conducted in four distinct settings: a tertiary hospital in Durban and three PHC centres across KZN, comprising two urban facilities and one in a township. The tertiary institution was selected based on its status as a prominent CKD treatment and management centre within the KZN region. Three PHC centres were strategically chosen to gather comprehensive data from diverse healthcare professionals treating patients from urban and rural areas.

### Population

The study population was many people with specific characteristics, consisting of healthcare professionals who were based at a tertiary institution in the eThekwini metropolis in KZN and three PHC centres in KZN. This assisted the researcher in gaining a broader perspective from participants. Inclusion criteria were the following: participants over the age of 18 years who were healthcare workers from a tertiary healthcare facility that offers kidney dialysis and work-up for the CKD programme (Institution A), and healthcare professionals from three PHC centres (Institution B, C and D). The researcher used this population in the study to describe the perceptions of these healthcare professionals on the management measures of CKD in KZN and to explore the guidelines adopted in managing patients with CKD at PHC. Healthcare professionals included doctors, nurses, social workers, psychologists and dieticians. Upon completion of the interview, respondents did not provide any additional information.

### Data collection

The researcher developed a semi-structured interview guide with open-ended questions to interview healthcare professionals. This allowed the researcher to clearly describe healthcare professionals’ perceptions of managing CKD at the PHC level in KZN. Strategies to enhance trustworthiness (rigour) in qualitative studies were employed throughout the research process. Through purposeful sampling procedures, participants and sites were identified to extract imperative information to understand the central phenomenon. Engagement with prospective participants was initiated before data collection commenced; the researcher communicated and scheduled appointments with the area managers before the actual interviews to ensure that data collection did not impact patient care. The researcher ensured that the participants were equipped with sufficient information about the nature of the study, allowing them to make informed decisions (informed consent form).

Data collection took place over 3 months. Each interview lasted 20 mins to 30 mins, and field notes were taken simultaneously to capture non-verbal cues. Coronavirus disease 2019 (COVID-19) protocols were strictly followed throughout the data collection process. A face mask was worn at all times during interactions, and hand sanitiser was available during the interview sessions. Interviews and questionnaires were conducted individually, not in groups, and all took place in a well-ventilated room. The researcher obtained detailed participant accounts, ensured the safe storage of all data documents and provided verbatim extracts to include participant voices in the data presentation while maintaining access and confidentiality. Data collection continued until saturation was reached.

### Data analysis

The qualitative data was analysed using open coding and conventional content analysis. This method was chosen to reduce the large amount of data gathered and make it more manageable while maintaining fidelity to the essential contents. Coding is a process of breaking down a large amount of information into categories, which can then be compared, conceptualised, examined and categorised (Cohen, Manion & Morrison 2002).

To identify codes, the researcher used a mind map to note key phrases and statements from the data. This process was applied to all transcripts. The identified codes were then organised into categories and subcategories, with all data grouped for further analysis to assign meaning. The researcher repeatedly reviewed the raw data to ensure accurate representation of participants’ voices and to fully understand the collected information (Cohen et al. 2002).

Subsequently, detailed descriptions were crafted for each category and subcategory, facilitating their organisation into overarching themes. The researcher further discussed these themes with supervisors, who cross-checked the emerging themes as part of quality assurance (Cohen et al. 2002). Vital information was also extracted from the transcripts.

### Ethical considerations

Authority to conduct this research was sought from the Head of the KwaZulu-Natal Department of Health at the head office in Pietermaritzburg via the Health and Research Ethics Committee. The researcher gained entry to the research settings and commenced data collection after obtaining full ethical approval from the University of KwaZulu-Natal Biomedical Research Ethics Committee (BREC/00004880/2022) and Municipal sites.

## Results

This study delves into healthcare professionals’ perspectives on interventions related to managing CKD in the PHC settings in KZN, in institutions A, B, C and D. Table 1 provides a breakdown of the categories of healthcare professionals included in the study. The analysis identified three overarching themes, four categories and four subcategories (Table 2).

**TABLE 1:** Demographic overview of healthcare workers.

Job title	Institution A	Institution B	Institution C	Institution D
Doctor	2	2	1	-
Nurse	3	4	3	2
Psychologist	1	-	-	-
Dietician	1	-	-	-
Social worker	1	-	1	-

**TABLE 2:** Themes, categories and subcategories of healthcare workers’ perceptions of chronic kidney disease management.

Themes	Categories	Subcategories
1. Knowledge and awareness about existing guidelines	Awareness of existing guidelines	Implementation of current guidelines
	Lack of knowledge about existing guidelines	Do staff possess the required knowledge to implement current guidelines?
2. Fragmented system and lack of clear guidelines	Uncoordinated care	Clarity of roles
3. Implications of clear guidelines on patient care	Clear guidelines for improving patient outcomes	Importance of clear guidelines on the work-up for patients into the CKD programme

CKD, chronic kidney disease.

### Theme 1: Knowledge and awareness about existing guidelines

Doctors and nurses displayed varying degrees of awareness of guidelines for CKD management. While some were well-informed and implemented primary CKD management measures, others expressed uncertainty. Screening measures included the Jaccold Assessment Tools and regular health education on dietary modifications. Participants emphasised the need for more comprehensive guidelines tailored to PHC settings. The following excerpt explains a participant’s view:

‘I think as I say these guidelines on dialysis is already at tertiary service. So I think from the primary setting one should look at how much screening and prevention should be emphasised. Now, this is if you look at the guide, and maybe we, we need to look at it, although we cover some screening, just like a small chapter on that. So in terms of the, the portion that’s advocated is very, very small. So it’s not, it’s not addressing it adequately in terms of how do you screen now who should you screen and how frequently can you screen, so all of these need to happen? So that probably we need to have another section that we deal with us specifically? Yes, we have, because this is so bad, it’s the final diagnosis. So it doesn’t cover adequately the setting of especially in the clinics, in district hospitals, this is a really tertiary level of care. So secondary, tertiary, because dialysis you will get at maybe in a regional hospital, definitely, that you are trying to do so that is why we need another one, at least more comprehensive guidelines. A document that you have seems to be the primary aspects.’ (Institution A- Participant 4, Male, Doctor)

#### Awareness of existing guidelines

Participants referred to guidelines such as Kidney disease Improving Global Outcomes (KDIGO) and the American College of Physicians’ guidelines for CKD screening, risk stratification and treatment. Healthcare professionals’ discrepancies in adherence to and awareness of these guidelines existed. The following narrative explains a participant’s view:

‘Not that I am aware of but it does happen at district and regional.’ (Institution A- Participant 5, Male, Doctor)

#### Implementation of current guidelines

Participants highlighted the importance of regular screening for high-risk individuals and adherence to guidelines, utilising tools like the Essential Drug List (EDL) to guide their practices. The following extract supports and describes a participant’s view:

‘We conduct urine testing using the Jaccold assessment tool e.g assessment for edema, we measure the patients weight, We give dietary education, we do bloods, creatinine levels every 6 months for diabetic and hypertensive patients yearly, for HIV patients we monitor them 3 monthly for if they on anti-retro viral treatment.’ (Institution B- Participant 3, Female, Nurse)

#### Lack of knowledge about existing guidelines

Some participants needed to be made aware of existing guidelines, indicating a need for ongoing education and awareness programmes to improve understanding among primary caregivers. The following narrative explains a participant’s view:

‘There are no guidelines formally, but information is transmitted through interaction at these hospitals through outreach.’ (Institution A- Participant 5, Male, Doctor)

#### Do staff possess the required knowledge to implement current guidelines?

Some participants needed to be equipped with the knowledge to implement current guidelines, whereas a few understood their role in the PHC setting. The following narrative explains a participant’s view:

‘Urinalysis- creatinine, eGFR < 60 we do Jaccold assessment, any symptoms e.g flank pain, we do a full assessment and refer to the doctor.’ (Institution D- Participant 1, Female, Nurse)

### Theme 2: Fragmented system and a lack of clear guidelines

Participants expressed concerns about the need for clear guidelines for CKD management in PHC settings. Views on services varied, with some emphasising the need for written guidelines to clarify referral processes. The following verbatim quote explains a participant’s view:

‘There’s nothing actually written down for me to say who I refer to do you understand so I will really appreciate something like that. Erm ya.’ (Institution D- Participant 2, Female, Nurse)

#### Uncoordinated care

Participants reported conducting screenings, blood tests, health education and dietary interventions. However, a lack of clarity on roles and responsibilities hindered effective patient management. The following extract explains a participant’s view:

‘We do urea and creatinine bloods, health education and dietary education.’ (Institution C- Participant 1, Female, Nurse)

#### Clarity of roles

Participants had varying views, some participants reported that CKD should be treated at tertiary institutions were dialysis treatment is available, where as other participants understood that screening and monitoring is part of the PHC annual practice guidelines and they were implementing these measures. The following narratives explains participant’s view:

‘“Provided that the patients are treated initially at a tertiary or regional level, and they have sufficient results, ultra sound blood results erm echo’s”, ECGS. Then possible the guidelines will assist us.’ (Institution C-Participant 2, Male, Doctor)

### Theme 3: Implications of clear guidelines on patient care

Participants recognised the potential benefits of updated guidelines in improving patient care and outcomes. The importance of early detection, lifestyle modifications and comprehensive guidelines for managing CKD in PHC settings was underscored. Participants had an equivalent view that updated guidelines will improve patient care. The following narrative supports the above statement:

‘The sooner the patient is caught it will be better for the patients, family, PHC and hospital, and a much improved lifestyle for the patient, especially the youth, nutrition programmes, lifestyle and dietary needs.’ (Institution B- Participant 3, Female, Nurse)

#### Clear guidelines for improving patient outcomes

Healthcare professionals emphasised that updated guidelines would aid in prompt identification, timely intervention and improved patient outcomes, especially when dealing with high-risk populations. The following narrative supports the above statement:

‘If they able to detect the stages earlier, if they monitoring process will be more efficient and educating the community about identifying risk factors that will definitely reduce the rate at which the stages advances.’ (Institution A- Participant 6, Female, Psychologist)

#### Importance of clear guidelines on the work-up for patients into the chronic kidney disease programme

Participants highlighted the need for early referrals, evidence-based treatment modalities and improved patient care services. Clear guidelines were essential for reducing mortality, easing the burden on tertiary facilities and enhancing the overall quality of care. The following narrative supports the above statement:

‘“Obviously” it’s going to have err, that will mean for me, more referrals obviously err because I feel that we keep some of the patients they stay in our system for some time and some of them are supposed to be referred earlier and then also then patients will have a better outcome if they are referred early.’ (Institution D- Participant 2, Female, Doctor)

## Discussion

This study sheds light on the nuanced perceptions of healthcare professionals regarding CKD management in PHC settings, emphasising the need for comprehensive and clear guidelines to enhance patient care and outcomes. While written guidelines on the Department of Health and WHO website and in peer-reviewed publications are the norm, other formats (e.g. videos available on platforms like YouTube) are often more readily accessible and, hence, more widely used. It would be beneficial if the Department of Health could use such platforms to make more information accessible to healthcare professionals and patients. This may bridge the gap between healthcare professionals being unaware of explicit guidelines for managing CKD in PHC settings.

Staff shortages in the healthcare sector have created significant challenges, particularly in specialised areas. The study’s sample size (*N*) was too small to capture the specific preferences, needs, and attitudes of different healthcare professionals, such as doctors, nurses, and psychologists. As a result, the recommendations provided are general and focus on the importance of CKD screening, prevention, and management, aligning with standard medical practices rather than being derived from specific findings of this limited study.

### Limitations

The study was conducted in public institutions from only one province, so the findings cannot be generalised to all healthcare professionals managing CKD patients. Due to staff shortages, the researcher was able to recruit only a limited number of participants from various disciplines at PHCs and tertiary institutions. Many healthcare professionals were unavailable, as some were absent for months during the COVID-19 pandemic. Additionally, as a member of a multidisciplinary team working with healthcare professionals, the researcher’s presence may have influenced participants’ responses to follow-up questions during the interviews. To minimise bias and reduce influence, the researcher employed bracketing during the interviews.

### Recommendations

Based on the findings presented in the ‘Results’ section, here are some recommendations to enhance the management of CKD in PHC settings in KZN:

1. Guideline development and dissemination
  Participants expressed the need for new guidelines, hence it is advised that comprehensive, contextualised and unambiguous guidelines for managing CKD at the PHC level be developed. Ensure these guidelines are easily accessible and distributed to healthcare professionals in a user-friendly format.
2. Training and education
  Because of various healthcare professionals lacking insight into current CKD management guidelines, there is a need to implement regular training sessions and educational programmes for healthcare professionals, focussing on the awareness and implementation of CKD management guidelines. This should address the varying levels of knowledge among healthcare professionals and improve overall understanding.
3. Inter-institutional collaboration
  During the study, it was discovered that certain healthcare professionals were unclear on the referral pathway and when to refer patients or who to refer to. Therefore, frequent outreach programmes and meetings can be scheduled to foster collaboration and communication between healthcare institutions (A, B, C and D) to establish a standardised approach to CKD management. Encourage sharing best practices and experiences to enhance the overall quality of care.
4. Roles and responsibilities clarification
  A gap in ambiguous roles was found in the PHC context, where certain healthcare professionals were unclear about their responsibilities. The tasks and responsibilities of healthcare professionals across different levels of care can be accurately stated and communicated by department heads. To ensure a smooth transition of healthcare services from PHC to tertiary levels, a carefully planned referral system must be established.
5. Utilisation of screening tools
  The regular use of screening tools, such as the Jaccold assessment tool, for early detection of CKD is advised because some healthcare professionals were unsure of which instruments may be utilised to test CKD patients in the PHC environment. Resources and training to ensure healthcare professionals are proficient in using these tools and interpreting results should be provided.
6. Community awareness programmes
  Community awareness campaigns should be implemented to educate the public on the symptoms, risk factors, and importance of early detection of CKD, as the disease’s early stages go unrecognised until difficulties are discovered in its later stages. This can contribute to preventing CKD and encourage individuals to seek healthcare services proactively.
7. Regular review and updates
  Healthcare professionals reported that they were not aware of any changes to the current recommendations for managing CKD. Therefore, it is important to establish a mechanism for regularly reviewing and updating CKD management guidelines to incorporate the latest evidence-based practices. This ensures that healthcare professionals have the most current information in their daily practice.
8. Promotion of multidisciplinary teams
  Because of a lack of clarity on clearly defined roles, the formation of multidisciplinary teams involving doctors, nurses, dieticians, psychologists and social workers to provide holistic care for CKD patients is encouraged. Clearly define the roles of each team member to ensure coordinated and comprehensive patient management.
9. Monitoring and evaluation
  The study revealed shortcomings in the execution of current CKD management guidelines; frequent audits and evaluations may help pinpoint issues and ascertain how the implementation of guidelines affects patient outcomes. In addition, it is important to implement a system for monitoring and evaluating adherence to CKD management guidelines.
10. Resource allocation
  Participants expressed that the PHC setting lacked adequate resources, and the researcher discovered inadequate staffing during the study. In order to facilitate efficient CKD management in PHC settings, department heads should push for sufficient resources, such as personnel, tools and drugs. It is important to ensure that healthcare facilities have the necessary resources to implement guideline recommendations.

By addressing these recommendations, healthcare systems in KZN can enhance the quality of CKD management, improve patient outcomes and contribute to the community’s overall well-being.

## Conclusion

The study has uncovered critical insights into the challenges and opportunities surrounding the management of CKD in the PHC settings in KZN. The findings underscore the pivotal role that updated guidelines can play in facilitating healthcare professionals’ prompt identification of individuals with CKD, enabling timely interventions to curtail disease progression, reduce complications and mitigate the associated cardiovascular risks.

The identified themes, namely knowledge about existing guidelines, fragmented systems’ lack of clear guidelines and implications of updated guidelines on CKD management, shed light on the multifaceted nature of CKD care. The unique medical support required for this patient group necessitates a holistic approach from healthcare professionals. Tailoring interventions to the specific needs of CKD patients is paramount for optimal outcomes.

However, a concerning observation emerges from the results. Despite the high incidence of CKD in South Africa, a significant proportion of healthcare professionals demonstrate uncertainty and a need for more precise insight into guidelines associated with CKD management at PHC level. This knowledge gap underscores the urgent need for targeted educational initiatives, comprehensive guideline development and inter-institutional collaboration to empower healthcare professionals with the necessary tools and information to address the growing CKD burden effectively.

In conclusion, the study advocates for a concerted effort to bridge the knowledge gap, promote guideline adherence and establish a cohesive and informed healthcare system that can adequately meet CKD patients’ unique needs in PHC settings and communities. Only through such collaborative and proactive measures can the healthcare community in KZN optimise CKD management, enhancing patient outcomes and lessening the toll that this chronic illness takes on patients and the more extensive healthcare system.
